# The Current Role of Salvage Surgery in Recurrent Head and Neck Squamous Cell Carcinoma

**DOI:** 10.3390/cancers10080267

**Published:** 2018-08-10

**Authors:** Marc Hamoir, Sandra Schmitz, Carlos Suarez, Primoz Strojan, Kate A Hutcheson, Juan P Rodrigo, William M Mendenhall, Ricard Simo, Nabil F Saba, Anil K D‘Cruz, Missak Haigentz, Carol R Bradford, Eric M Genden, Alessandra Rinaldo, Alfio Ferlito

**Affiliations:** 1 Department of Head and Neck Surgery, King Albert II Cancer Institute, St Luc University Hospital, 1200 Brussels, Belgium; sandra.schmitz@uclouvain.be; 2 Institut de Recherche Expérimentale et Clinique (IREC), Université Catholique de Louvain, 1200 Brussels, Belgium; 3 Instituto de Investigación Sanitaria del Principado de Asturias and CIBERONC, ISCIII, Instituto Universitario de Oncología del Principado de Asturias, University of Oviedo, 33011 Oviedo, Spain; csuareznieto@gmail.com; 4 Department of Radiation Oncology, Institute of Oncology, Ljubljana SI-1000, Slovenia; pstrojan@onko-i.si; 5 Department of Head and Neck Surgery, The University of Texas MD Anderson Cancer Center, Houston, TX 77006, USA; KArnold@mdanderson.org; 6 Department of Otolaryngology, Hospital Universitario Central de Asturias, IUOPA, University of Oviedo, CIBERONC, 33011 Oviedo, Spain; jprodrigo@uniovi.es; 7 Department of Radiation Oncology, University of Florida, Gainesville, FL 32610-0385, USA; mendwm@shands.ufl.edu; 8 Head and Neck Cancer Unit, Guy’s and St Thomas’ Hospital NHS Foundation Trust, London SE1 9RT, UK; Ricard.Simo@gstt.nhs.uk; 9 Department of Hematology and Medical Oncology, The Winship Cancer Institute of Emory University, Atlanta, GA 30305, USA; nfsaba@emory.edu; 10 Head Neck Services, Tata Memorial Hospital, Parel Mumbai 400012, India; docdcruz@gmail.com; 11 Division of Hematology/Oncology, Department of Medicine, Morristown Medical Center/Atlantic Health System, Morristown, NJ 07960, USA; Missak.Haigentz@atlantichealth.org; 12 Department of Otolaryngology—Head and Neck Surgery, University of Michigan, Ann Arbor, MI 48109, USA; cbradfor@med.umich.edu; 13 Department of Otolaryngology—Head and Neck Surgery, Mount Sinai Medical Center, New York, NY 10029, USA; Eric.Genden@mountsinai.org; 14 University of Udine School of Medicine, 33100 Udine, Italy; alessandra.rinaldo@uniud.it; 15 International Head and Neck Scientific Group, 35030 Padua, Italy; alfio.ferlito@uniud.it

**Keywords:** cancer recurrence, head and neck cancer, squamous cell carcinoma, treatment failure, salvage surgery

## Abstract

Chemoradiotherapy has emerged as a gold standard in advanced squamous cell carcinoma of the head and neck (SCCHN). Because 50% of advanced stage patients relapse after nonsurgical primary treatment, the role of salvage surgery (SS) is critical because surgery is generally regarded as the best treatment option in patients with recurrent resectable SCCHN. Surgeons are increasingly confronted with considering operation among patients with significant effects of failed non-surgical primary treatment. Wide local excision to achieve clear margins must be balanced with the morbidity of the procedure, the functional consequences of organ mutilation, and the likelihood of success. Accurate selection of patients suitable for surgery is a major issue. It is essential to establish objective criteria based on functional and oncologic outcomes to select the best candidates for SS. The authors propose first to understand preoperative prognostic factors influencing survival. Predictive modeling based on preoperative information is now available to better select patients having a good chance to be successfully treated with surgery. Patients with a high comorbidity index, advanced oropharyngeal or hypopharyngeal primary tumors, and both local and regional recurrence have a very limited likelihood of success with salvage surgery and should be strongly considered for other treatments. Following SS, identifying patients with postoperative prognostic factors predicting high risk of recurrence is essential because those patients could benefit of adjuvant treatment or be included in clinical trials. Finally, defining HPV tumor status is needed in future studies including recurrent oropharyngeal SCC patients.

## 1. Introduction

Treatment with concomitant chemoradiotherapy (CRT) has emerged as a gold standard in the majority of advanced squamous cell carcinoma of the head and neck (SCCHN) [1,2]. The presumed advantages of CRT over radical surgery are organ preservation, better functional outcome, and a lower rate of acute complications. Compared to radiotherapy (RT) alone, the addition of chemotherapy (CT) delivered concomitantly with RT improves the survival of patients with advanced SCCHN with an overall 4% benefit at five years and greater benefit (8%) observed when CT was administrated concomitantly to RT [2]. However, because approximately 50% of the patients treated for Stage III and IV disease relapse after nonsurgical primary treatment [1,2,3,4], the role of salvage surgery (SS) is critical because often, it is the only remaining treatment option with curative intent. Surgery for patients with recurrent SCCHN is challenging. SS requires experienced surgical teams with expertise in reconstruction of complex surgical defects using multiple types of flaps. In the head and neck region, wide local excision to achieve clear margins must be balanced with the morbidity of the procedure, the functional consequences of organ mutilation, and the likelihood of success. The extent of surgery required to resect the tumor with clear margins is often difficult to assess because the precision of histological margins of recurrent tumors is troublesome to accurately delineate. The combination of treatment toxicity and patient’s comorbidities lead to a higher rate of postoperative complications. These side effects must be balanced against chances of cure so that the most suitable candidates may be offered surgical salvage. In view of all these factors, it is essential that multidisciplinary teams involved in the management of these patients establish criteria based on functional and oncological outcomes to select the best candidates for SS. SS requires an open interactive discussion with the patient and relatives to provide optimal care, based on objective information on outcome prospects. In this respect, physicians should keep in mind that the benefits of cure do not always justify excess morbidity with poor quality of life and must take strongly into account the individual patient’s condition and preferences.

To better select the best candidates for SS, the authors propose first to understand perioperative prognostic factors influencing survival. Then, after completion of SS, one must identify patients with postoperative prognostic factors predicting high risk of recurrence who could benefit of adjuvant treatment or be included in clinical trials. Finally, toxicity and survival trade-off must be considered.

## 2. Accurate Selection of Patients for Salvage Surgery

The emergence of organ preservation strategies has dramatically limited the role of primary surgery in advanced SCCHN. However, despite combinations of CT and RT, 40% to 60% of patients with advanced-stage tumors still relapse [4]. Because surgery is generally regarded as the best treatment option in patients with recurrent resectable SCCHN, surgeons are increasingly confronted with considering operation among patients with significant effects of failed non-surgical primary treatment. SS remains associated with high morbidity and poor oncologic outcomes [5]. Besides the surgeon’s expertise, accurate selection of the patients suitable for surgery is a major issue. Not all patients are suitable candidates for SS. Accordingly, objective criteria to accurately select patients who are the best candidates for salvage surgery is of paramount importance to facilitate decision making in patients with tumor recurrence.

Prior RT with or without CT influences wound healing and increases the risk of wound complications. SS requires often large resections performed on poorly vascularized hypoxic and fibrotic tissues [6]. Because CT is most commonly employed in conjunction with RT, it is difficult to delineate its independent impact. As a sensitizer, CT likely compounds the cellular damages of radiation. Additionally, CT may have a negative impact on the systemic medical condition of the patients [7].

## 3. Preoperative Optimization

Factors such as low performance status and poor general medical condition often lead to wound complications after SS. Strategies to reduce the rate of local complications include preoperative optimization of known wound healing factors, adjunctive wound care modalities, and the use of distant flaps carrying a significant amount of well vascularized tissue. Factors such as nutrition, blood glucose control, smoking, and alcohol abuse can be optimized prior to surgery. Nutrition referral and methods to optimize preoperative nutrition should be routine. Controlling blood glucose level, managing immunosuppressant medications, optimizing thyroid hormone levels, and improving pulmonary function are factors that can be optimized preoperatively to promote healing postoperatively [7]. Preoperative functional assessment should be considered among patients planned for organ sparing SS to understand the pre-existing impact of non-surgical therapy, need for longer term enteral nutrition, and counsel appropriately about reasonable functional expectations and rehabilitation potential after SS.

## 4. Peroperative Use of Flaps

Because reconstruction in SS should be performed with fresh vascularized tissue, the use of distant flaps is strongly recommended with microvascular free tissue transfer as the gold standard. In reported series of patients who have had salvage procedures for recurrent SCCHN, the rate of free flaps and pedicled flaps performed to repair surgical defects ranges from 52% to 56% [3,8,9].

## 5. Awareness of Complications in Salvage Surgery

While it is difficult to compare data across trials in the absence of well accepted guidelines, reported rates of complications following SS for all head and neck subsites ranged from 23% to 67% [3,8,9,10,11,12,13,14,15,16,17]. In the absence of uniformity in the reporting of complications, comparison is challenging. Many authors report all complications [9,15] whereas other authors report only major complications [16] or local complications [8]. Currently, the classification system of postoperative complications proposed by Dindo and Clavien is becoming widely accepted and records all postoperative complications data [18]. This system, commonly used in general surgery, was recently adopted for head and neck surgical oncology [19]. Prospective studies should adopt a single classification system widely accepted by head and neck surgeons.

A review of the complications following total laryngectomy after (C)RT failure including 3293 patients reported a rate of complications of 67.5%. Pharyngo-cutaneous fistula was the commonest complication with a pooled incidence of 28.9% [20].

Patients with SCCHN who had surgery for the primary tumor with concomitant neck dissection (ND) are at higher risk of complications than patients who underwent surgery for the primary tumor or ND alone. As reported in primary surgery, ND has been identified as an independent factor related to complication rate of 33–67.5% in patients who had salvage ND in combination with primary tumor resection [19,20,21,22]. Typically, salvage ND is performed for more advanced recurrent disease, requires wider incisions, increases operating time, and exposes many neurovascular structures at risk for injury. It has been reported that the occurrence of surgical complications was an independent predictive factor for poor prognosis after SS following CRT [16].

## 6. Oncological Outcomes

Many studies have identified advanced primary tumor and nodal stage, short disease-free interval (DFI), nonlaryngeal cancer site, and previous RT as oncologic risk factors for poor outcome after SS [9,17,23,24,25,26].

A comparison of oncologic outcomes between reported studies is challenging (Table 1). Some studies address salvage treatment for specific subsites (e.g., oral cavity and oropharynx, larynx only, larynx and hypopharynx, oropharynx only, etc.) [11,13,14,15,17,27,28] while others analyze the results of all salvage modalities pooling together surgery and non-surgical salvage modalities [23] or report results after failure of a specific primary treatment like CRT [3,16]. In a study addressing SS for all head and neck sites, Hamoir et al. reported a two-year overall survival (OS), disease-specific survival (DSS) and disease-free survival (DFS) rates of 59%, 66%, and 56% respectively and five-year OS, DSS, and DFS rates of 42%, 56.5%, and 47%, respectively [9]. These results are in line with the results of previously reported series [3,8,15]. In a series reported by Tan from Institut Gustave Roussy (Villejuif) of 93 patients who experienced failure after CRT, 38 patients had SS whereas 55 patients were treated with palliative intent [3]. Median OS for the salvage group was 19.4 months compared with 4.3 months for the palliative group. Two- and five-year survival rates for the former were 43.4% and 36.5%, respectively whereas one- and two-year survival rates for the latter were 10.6% and 0%, respectively. Esteller et al. reported a five-year survival rate of 34.2% after SS [8]. Van Der Putten et al. reported a five-year OS of 27%, in a series of patients who underwent SS for recurrent laryngeal and hypopharyngeal cancers [15]. Goodwin reported a five-year OS of 39% in a large multicenter retrospective series of 1080 patients with recurrent tumors from all head and neck sites [5]. Taguchi et al. reported a five-year OS of 61% for patients with recurrent oropharyngeal, laryngeal, or hypopharyngeal cancer who had SS after CRT [16]. However, SS was performed in only one-third of patients with recurrent disease and, of those, one-third had ND alone, suggesting stringent selection criteria.

Taguchi et al. found that initial Stage IV disease, poorly differentiated histology, and synchronous second primary cancer, were significant predictors of unfavorable OS on multivariate analysis [16]. Tan et al. found that initial Stage IV tumors and concurrent local and regional failure were independent predictors for poor survival after SS [3].

### 6.1. Impact of the Anatomical Site on Outcome

Oropharyngeal and hypopharyngeal primary sites, advanced primary tumor stage and locoregional recurrence are reported as preoperative independent prognostic factors for reduced survival. Laryngeal recurrence is associated with more favorable survival outcomes relative to oropharynx and hypopharynx sites. In patients with recurrent laryngeal cancer following RT or laser microsurgery, salvage total and supracricoid laryngectomy enable wide resection with clear margins in most patients and offer five-year OS rates ranging from 57% to 70% [9,28,29,30].

Although oral cavity recurrences may be early detected, survival rates are lower than those observed for patients with laryngeal SCCs. Oral cavity recurrences have been reported to be equally likely to occur among local, locoregional, and regional sites. Additionally, distant metastases are not infrequently observed [31]. On the other hand, patients with recurrent laryngeal cancer are typically treated by primary RT while many patients with recurrent oral SCC are primarily treated with surgery alone and are therefore candidates for adjuvant RT or CRT following surgical salvage. Matsuura et al. recently reported a series of 46 patients with oral SCC primarily treated by surgery alone (33/46) or followed by RT or CRT who underwent SS for local or local and regional recurrence. After SS, 13 patients (28.3%) received adjuvant treatment. With a median follow-up time of 18 months and a maximum of 61 months, 27 of 46 patients (58.7%) had a second disease recurrence. Six patients had only a local recurrence, 5 had only regional disease, 15 had combined local and regional recurrences, 9 had distant metastasis (one of which was isolated), and 23/46 (50%) died during follow-up. OS, DSS DFS rates were 31.7%, 36.2%, and 35.0% respectively. The presence of lymph node metastasis and positive surgical margins were the only independent factors associated with both recurrence rates and mortality [32]. In a series of 185 patients treated for recurrent oral SCC, (17 patients were excluded from analysis because developing recurrence within six months after completion of the primary treatment), the five-year OS rate was 31.9%. A significant difference was noted in the five-year OS rate between patients with local alone vs. local and regional recurrence (37.5% vs. 21.5%). Patients with relapse more than 18 months after completion of their primary treatment had significantly improved OS rates compared with those who relapsed within 18 months of initial treatment (42.3% vs. 20.5%) [33]. In a series of 528 patients with recurrent oral SCC, Liu et al. reported a five-year OS of 31.5%. Patients with recurrence interval <18 months had a lower probability of survival than those with recurrence interval ≥18 months (27.6% vs. 38.2%, respectively) [34].

Results after SS for recurrent oropharyngeal cancer are generally disappointing with survival rates ranging from 13% to 31% at five years [9,13,14,17]. The anatomy of the oropharynx and its proximity to the skull base leads to difficulties achieving clear margins in advanced recurrent tumors [11] resulting in low five-year DFS rates of 19–27% [9,17]. In a study from the MD Anderson Cancer Center (Houston), reporting results of salvage treatments in recurrent oropharynx cancer, patients treated with SS had a five-year OS of 28% [14].

The survival of patients with recurrence in the hypopharynx is poor. Hamoir et al. reported that patients undergoing SS for a recurrent hypopharyngeal SCC had a five-year OS of 15.5% [9]. Salvage laryngopharyngectomy often combined with esophagectomy carries a high risk of major wound complications and perioperative mortality [35,36]. Recurrent hypopharyngeal cancers are frequently not resectable [35]. This underscores the need to realistically frame the severe functional consequences of SS associated with a lower rate of cure. Nevertheless, when oncologically sound, SS remains the best available treatment to control disease and improve survival. In patients undergoing salvage surgery for recurrent hypopharyngeal cancer, five-year DFS rates of 40–50% have been reported from experienced tertiary centers [9,36,37].

### 6.2. Impact of the Initial Stage and Recurrent Stage on Outcome

Initial stage has been reported having an impact on prognosis. Investigators from the MD Anderson Cancer Center reported a series of 218 patients who underwent salvage total laryngectomy where 70% of patients had initial early T1-T2 N0 tumors. The five-year disease control and OS rates were 65% and 57% respectively [28]. Hamoir et al. have reported that patients with initially advanced stage tumors were six times more likely to develop recurrence compared to patients with Stage I tumors [9]. Many other authors have reported decreased survival rates in patients with advanced initial tumors because of a higher likelihood of locoregional recurrence and distant metastasis after SS [3,15,16,17,38].

Extent of disease at the time of recurrence is also of substantial importance in predicting prognosis [5,11,38,39]. In a prospective study including 109 patients, Goodwin [5] identified a relevant difference in two-year DFS based on recurrent stage: 67% for rStage II vs. 33% for rStage III and 22% for rStage IV, underscoring the difficulty of successful treatment in patients with advanced recurrent disease. In contrast, some have not observed this relationship [9,38].

### 6.3. Impact of the Disease-Free Interval on Outcome

DFI is defined as the interval between the end of the first treatment until evidence of recurrence. It has been reported that a short DFI had a significant negative prognostic impact [14,40,41]. A period of six months posttreatment is often proposed as the threshold to define persistent disease vs. tumor recurrence. Patients with tumor present within six months at the end of treatment were considered as having persistent disease. Some authors have proposed a threshold of up to two years [17,27,42,43,44] while a period of six weeks only was proposed in patients treated with RT alone [14]. In series of patients with recurrent oral SCC, those relapsing <18 months after completion of their primary treatment had a lower probability of OS than those with an interval ≥18 months (20.5% vs. 42.3% and 27.6% vs. 38.2% respectively) [33,34]. However, in one of those studies, patients developing recurrence within six months after completion of the primary treatment were excluded from analysis [33]. Additionally, it should be emphasized that most recurrences are observed within the first 18 months following treatment of the primary tumor. These various differences in defining DFI thresholds could explain contradictory results between studies. Overall, it seems logical to consider that patients with persistent tumor at the time of the posttreatment assessment (typically, 12 weeks after completion of RT or CRT) should be considered as having persistent disease.

## 7. Survival Predictor Scores

To better select patients for SS, Tan et al. developed a model based on data available before salvage, stratifying patients into prognostic groups that predict postsalvage survival. Initial Stage IV tumors and concomitant local and regional failures were independent predictors of decreased survival [3]. Two-year OS rates for patients with none, one or two of these predictive factors were 83%, 49%, and 0%, respectively, suggesting that patients with initial Stage IV and concurrent local and regional failure should not be candidates for SS. In contrast, Esteller et al. were not able to find any significant difference in survival when analyzing their patients according to this model [8]. Hamoir et al. proposed another survival predictive score incorporating three independent preoperative predictors: local and regional failure, tumor site (larynx vs. non-larynx), and initial stage (Stage I/II vs. Stage III/IV). Patients with none, one, two, and three predictive factors of outcome had successful salvage rates of 96.2%, 62.5%, 35.5%, and 28.6% respectively [9]. According to this predictive score, patients with more than one predictor had limited chance to be successfully treated by SS. Such survival predictive scores should be helpful for clinicians to better decide whether or not to proceed with SS, taking into account the functional consequences (voice, swallowing) and potential morbidity, and to objectively counsel patients facing recurrent SCCHN and its subsequent management decisions.

## 8. Identification of Patients at High Risk of Re-Recurrence after Salvage Surgery

### 8.1. Impact of Margins and Extracapsular Spread

After SS has been performed, it is essential to identify patients who are at high risk for a second recurrence and who are possibly candidates for adjuvant therapy. Resection margin status and extracapsular spread (ECS) are significantly related to survival following SS after CRT [3,8,43]. Many studies have identified positive margins as a risk factor of poor oncologic outcome [9,23,24,25,26,32,40]. The presence of positive margins after SS, has been reported in 18.3–22% of patients [3,9] and it is a strong independent factor for recurrence in many studies [9,14,32]. Nichols et al. reported a five-year OS of 43.4% when complete resection of the tumor was achieved. The ability to obtain negative margins was independently associated with improved survival (*p* = 0.001) [27].

However, other were not able to find a significant correlation between margins and survival [14,17], highlighting the difficulty in accurately assessing margins in previously irradiated tissues. This has been demonstrated by Jones in a study including 352 SCCHN patients treated by SS after RT. The five-year failure rate at the primary site was 47% for those with negative margins and 66% for those with positive margins (*p* = 0.0286). The five-year DSS of patients with positive margin was 31% vs. 43% for those patients with negative margins (*p* = 0.022) [45]. Of note, patients with negative but close margins were also reported to be at higher risk for developing a second recurrence [9].

### 8.2. Impact of Complications in Outcomes

The occurrence of postoperative complications was reported to be correlated with poor oncologic outcomes [9]. Postoperative complications occur more frequently in patients with comorbidities, tobacco and alcohol addiction, and/or malnutrition [7,21,22,46]. Analyzing only local complications, Esteller et al. did not find any correlation between the occurrence of complications and age, primary site, reconstruction, initial stage, and prior CT [8]. According to Futura et al., there was a correlation between initial concomitant CRT and postoperative complications, especially pharyngo-cutaneous fistulas requiring surgical intervention for repair [47]. Hamoir et al. reported that patients undergoing surgery requiring reconstruction with a distant flap and surgery performed on the tumor site combined with ND were more likely to have postoperative complications. Overall, positive margins, ECS, and occurrence of postoperative complications were independent postoperative prognostic factors predicting for lower survival [9].

### 8.3. Other Key-Factors to Strongly Consider

In many studies addressing the role of SS for recurrent SCCHN, human papilloma virus (HPV) status of recurrent oropharyngeal SCC was not investigated. In general, HPV- positive patients have less extensive tumors in a healthier environment not compromised by alcohol and tobacco abuse. SS seems to be more beneficial in terms of survival in HPV-positive patients when compared with HPV-negative patients. The assumption that R0 margins were easier to obtain in recurrent oropharyngeal SCC [27] was not confirmed by others [48]. Another reason gaining importance in the future would be a higher sensitivity of HPV-positive oropharyngeal primaries to non-surgical therapies and, consequently, possibility of treatment de-intensification strategies which are presently under extensive evaluation. Reduced toxicity of such treatments increases chances for successful SS in case of recurrence.

Recently, different studies have tried to elucidate this issue. In a retrospective analysis of 108 patients with recurrent oropharyngeal SCC, Guo et al. (2015) reported that HPV-positive patients had significantly improved three-year OS after recurrence as compared with HPV-negative patients (55.7% vs. 25.2%). Additionally, patients who underwent SS had significantly improved three-year OS compared to patients who received non-surgical salvage (61.8% vs. 24.1%). HPV-positive tumor status, longer time to recurrence, primary surgical treatment, and treatment with SS were each independently associated with improved OS [48]. In accordance with these data, in a series of 86 recurrent oropharyngeal SCC the two-year OS rate was improved for HPV-positive as compared with HPV-negative patients (89.0% vs. 61.9%). Patients with HPV-positive tumors had a statistically significant reduction of risk of death and SS was similarly associated with a better OS [49]. These results are in line with recent publications reporting that HPV-positive patients with recurrence preserve their HPV-positive phenotype, not similar to HPV-negative patients [50,51].

Conversely, Sweeny et al. studied 69 patients who underwent SS for a recurrent oropharyngeal SCC after primary RT. A lower incidence of recurrence after SS was reported in HPV-positive tumor patients compared with HPV -negative tumor patients (28% vs. 52%). However, HPV-positive tumor patients and HPV-negative tumor patients had comparable two-year and five-year OS (51% and 27%) [52]. This is in agreement with Patel et al. (2016), who found no differences in the five-year DFS rates for HPV-positive patients compared with negative patients (21% vs. 12%) [53]. Thus, defining HPV tumor status is needed in future studies including patients with oropharyngeal SCC [48,49,50,51].

Age and performance status of the patients are important factors determining outcomes in patients with recurrent SCCHN. Kim et al. recently demonstrated that medical comorbidities and age must be strongly considered when selecting candidates for SS [42] as survival is adversely affected. They retrospectively reviewed 191 patients with recurrent SCCHN treated with SS with one-year OS after salvage as the primary endpoint. Charlson age comorbidity index (CACI) prior to salvage was calculated; 27.7% (53/191) of patients died within one year after SS. To evaluate the CACI as a predictor of mortality, OS survival curves were created after stratification of patients into two categories: CACI score ≥6 (112 patients) vs. <6 (79 patients), which revealed a significant difference in OS (*p* < 0.001). In multivariate analysis, for every unit increase in total CACI score, the odds of death within one year after SS increased by 43% while controlling for other factors. Primary T3-T4 tumors and DFI < 6 months were also significant independent predictors of one-year mortality. Patients who died within one year had more hospital admission, longer total length of hospital stay, higher risk of discharge to a skilled nursing facility, and spent 17.3% of their remaining days in the hospital. The mean CACI score was significantly higher for those with less than one-year OS compared with those with OS of one year or more (8 vs. 6, *p* = 0.001). Medical comorbidity and age as measured by the CACI, in addition to known factors must be considered in selecting patients candidates for SS. Patients with these risk factors should be more strongly considered for palliative measures [42].

Whereas SS remains the standard of care in patients with recurrent resectable SCCHN, it is not suitable in all patients because of local or general contraindications. In the MD Anderson Cancer Center study already mentioned, reporting outcomes of salvage treatments in recurrent oropharynx cancer, patients treated with palliative treatment had a five-year OS of 0%, compared to 28% after SS [14]. Investigators from Barcelona reported a series of 1088 patients with local and/or regional recurrent SCCHN. Half of them (562/1088) were suitable for salvage treatment. SS was performed in 89.5% (503/562) of those patients, and salvage RT or CRT in 10.5% (59/562). SS was associated with adjuvant RT or CRT in 22.5% (113/503) of the patients. Considering the bias inherent to any retrospective study, the five-year DSS of patients treated with salvage surgery was clearly higher than survival of patients treated with salvage RT/CRT (58.3% vs. 26.4%) [44]. Results comparing SS and reirradiation are frequently biased because patients treated with the latter often have incompletely resectable disease and would be more likely to experience poor outcomes regardless of treatment [31].

## 9. Role of Nonsurgical Treatment as Salvage and as Adjuvant Treatment

When SS is contraindicated, reirradiation may play a role in selected patients and encouraging five-year OS results of 32% have been reported in highly selected patients [14]. For patients deemed as having an unresectable recurrence or unfit for salvage SS, salvage RT in previously irradiated sites (targeting only the recurrent tumor site) has been reported, but with modest or poor results depending on patient selection and extent of disease [54]. Undoubtedly, reirradiation is a subject of even more restrictions than SS because of high toxicity, impaired quality of life, and limitations on reirradiation dose [55,56]. Previously irradiated target volumes and the proximity of vital structures limit the dose and volume that can be safely reirradiated and can result in significant toxicity, such as carotid blowout, osteoradionecrosis, radiation myelopathy, sepsis, and death [11,55,56]. A treatment mortality rate of 8% has been reported [56]. It has been reported that microvascular free flap reconstruction may reduce the toxicity of reirradiation following salvage surgery [7,57]. The high likelihood of severe complications and unclear survival advantage show that re-RT should be carefully considered prior to treatment patients who are not surgical candidates [31]. Intensity-modulated RT, stereotactic body RT, as well as proton beam RT have emerging roles in the treatment of selected patients, delivering higher and more conformal doses while minimizing irradiation of normal tissues [58,59,60].

When reirradiation is considered, if the patient can tolerate it, a chemotherapeutic agent—preferably to which the patient is naïve—should be administrated to improve the likelihood of tumor control. However, toxicity remains a major issue. Previous use of CT was identified as an adverse prognostic factors when intensive CRT was employed for salvage, most probably due to resulted more pronounced fibrosis (with creation of hypoxic environment and compromised drug delivery) and/or the presence of highly resistant tumor clonogens that survived previous (C)RT [61]. Two Radiation Therapy Oncology Group (RTOG) trials prospectively assessed the role of hyperfractionated reirradiation combined with CT in patients with recurrent unresectable SCCHN [62,63], with encouraging survival rates at two years. However, the incidence of grade 4–5 acute toxicity was high, with 8% to 9% treatment-related mortality. Of note, reirradiation studies have shown long-term OS rates of 36% to 60% when SS was initially performed, compared with 9% to 17% when surgery was not achieved [55,63,64].

The utility of postoperative reirradiation or combined chemoreirradiation following salvage surgery in patients primarily treated with RT remains questionable. In patients who had poor prognosis factors following SS, results from a randomized study have shown that DFS and locoregional control (but not OS) were improved when surgery was followed by concomitant CRT compared to surgery alone [65].

However, CT regimen was not widely used and toxicity was substantially increased. An 8% rate of treatment-related deaths was reported in the group of patients randomized to the chemoreirradiation arm and a 39% rate of late grade 3–4 toxicity was observed in patients surviving 24 months.

The emergence of immunotherapy may play an increasing role for patients with incompletely resectable disease. Recently, it has been reported that use of Nivolumab, a monoclonal antibody against the programmed-death-receptor-1 (PD-1) monoclonal antibody was associated with prolonged survival when compared with standard single-agent systemic therapy in patients with platinum-refractory recurrent SCCHN. Compared with standard treatment, Nivolumab was associated with a better toxicity profile and a better quality of life in those patients with recurring refractory tumors [66]. In the next weeks, a multicenter randomized phase II study will be opened comparing adjuvant immunotherapy plus standard follow-up vs. standard follow-up alone in high-risk patients with recurrent HNSCC treated with SS.

## 10. Conclusions

Because other effective options are still lacking, SS remains the treatment of choice whenever feasible in patients with recurrent SCCHN. However, it is important to understand which patients are most likely to benefit from SS. Predictive modeling based on preoperative information is now available to better select patients having a good chance to be successfully treated with SS. Patients with a high comorbidity index, advanced oropharyngeal or hypopharyngeal primary tumors, and combined local and regional recurrence are very unlikely to be treated successfully with salvage surgery and should be considered for other treatment options. This information is critical to decide whether or not to proceed with SS and to better counsel patients with recurrent SCCHN. After SS, patients with positive margins and ECS are at high risk for recurrence should be strongly considered for enrollment in clinical trials of adjuvant therapy. Future clinical trials of adjuvant immunotherapy following salvage surgery in high-risk patients are in progress.

This article reflecting the opinion of the authors was written by members and invitees of the International Head and Neck Scientific Group (www.IHNSG.com).

## Figures and Tables

**Table 1:** Reported overall survival rates in recent series of patients who had salvage surgery for recurrent head and neck squamous cell carcinoma.

Author	Patients (n)	Two-Year Overall Survival %	Five-Year Overall Survival %
Goodwin (2000) [5] ^1^	1080		39
Zafereo et al. (2009) [14] ^2^	134	34	28
Tan et al. (2010) [3]	41	43.4	36.5
Nichols et al.(2011) [27] ^2^	26	64	43
Esteller et al. (2011) [8]	32	40	34.2
Righini et al. (2012) [13] ^2^	105	31	21
Van Der Putten et al. (2015) [15] ^3^	22		27
Taguchi et al. (2016) [16]	78		61
Agra et al. (2016) [11] ^4^	246		32.3
Hamoir et al. (2017) [9]	109	59	42
Philouze et al. (2017) [17] ^2^	52	43	31

*n* = number of patients. ^1^ Meta-analysis combining 32 series including 1080 patients. ^2^ Oropharyngeal SCC only. ^3^ Laryngeal and hypopharyngeal SCC only. ^4^ Oral cavity and oropahryngeal SCC only.
